# Bridging regenerative medicine with digital intelligence: enhancing stem cell therapy for female infertility through AI and digital health

**DOI:** 10.1097/JS9.0000000000003086

**Published:** 2025-07-22

**Authors:** Jingxuan Peng, Sha Li, Zhengyan Tang

**Affiliations:** a Department of Urology, First Affiliated Hospital of Jishou University, Jishou, Hunan, China; b Department of Urology, Xiangya Hospital, Central South University, Changsha, Hunan, China; c Provincial Laboratory for Diagnosis and Treatment of Genitourinary System Disease, Changsha, Hunan, China; d Department of Burns and Plastic Surgery, Xiangya Hospital, Central South University, Changsha, Hunan, China

*Dear Editor,*

We read with great interest the comprehensive analysis by Jin *et al* detailing the global clinical trial landscape of stem cell therapy for female infertility[1]. The authors provide a crucial analysis highlighting the field’s exploratory phase, the dominance of early-phase trials (particularly in China), the prevalence of perinatal-derived stem cells like UC-MSCs, and the focus on conditions like primary ovarian insufficiency (POI) and intrauterine adhesions (IUA). While the study adeptly outlines current challenges—standardization, geographic generalizability, and the need for mechanistic insights—we wish to extend the discussion on a transformative dimension: the integration of artificial intelligence (AI) and digital health technologies to overcome these hurdles and unlock the full potential of stem cell therapies. And we ensure the article is compliant with the TITAN Guidelines[2].

The heterogeneity in cell sources, preparation methods, administration protocols, and patient responses identified by Jin *et al* creates a complex optimization problem perfectly suited for AI-driven solutions. Machine learning (ML) algorithms can analyze vast, multi-modal datasets encompassing patient genomics, proteomics, clinical history, detailed stem cell characterization (transcriptomics, secretome, potency assays), and treatment outcomes from both completed trials and real-world evidence. Such analysis can identify predictive biomarkers of therapeutic response and pinpoint specific stem cell subpopulations or derivatives (like exosomes) with maximal efficacy for distinct infertility pathologies (e.g., POI vs. IUA) or patient endotypes.

Furthermore, digital health platforms are essential for robust longitudinal monitoring and outcome assessment, crucial areas needing enhancement as highlighted by Jin et al. concerning unpublished results and the need for standardized follow-up. Wearable sensors and mobile health apps enable continuous, remote monitoring of key physiological parameters relevant to infertility treatment response, such as basal body temperature patterns, hormone levels (via emerging biosensors), menstrual cycle regularity, and patient-reported outcomes (PROs) like symptoms and quality of life. Integrating this rich, real-time data into electronic health records (EHRs) creates a dynamic dataset far surpassing traditional clinic visits. AI can then analyze these longitudinal streams to detect subtle signs of improvement or adverse events earlier, identify non-responders promptly, and correlate real-world outcomes with specific therapeutic protocols. As emphasized by Huckvale *et al*, such digital phenotyping enhances the precision and efficiency of clinical evaluation in complex therapies[3].

The aspiration towards personalized treatment regimens, mentioned by Jin *et al* as a future direction, can be accelerated by AI-powered clinical decision support systems (CDSS). These systems can synthesize individual patient data (clinical history, imaging, omics profiles, digital biomarkers) with the aggregated knowledge from global clinical trials and scientific literature. This synthesis can guide clinicians in selecting the optimal stem cell type, dose, route of administration (e.g., systemic vs. local injection), and potentially combinatorial strategies (e.g., with hormonal support or biomaterials) tailored to the patient’s specific infertility etiology and biological profile. Work by Hui Ting Ong *et al* demonstrated the feasibility of digitalized organoids, illustrating the path toward true personalization[4].

In conclusion, while stem cell therapy holds immense regenerative promise for female infertility, its path to clinical maturity faces significant challenges in standardization, efficacy prediction, and personalized optimization. The integration of AI for biomarker discovery, cell product characterization, and treatment personalization, coupled with digital health technologies for enhanced monitoring and data collection, offers a powerful synergistic approach. These technologies can directly address the critical gaps identified by Jin *et al*, paving the way for more effective, reliable, and individualized regenerative therapies for women worldwide[1]. Future clinical trials and research initiatives in this space should actively incorporate and evaluate these digital intelligence tools as integral components of stem cell therapy development and deployment.

## Data Availability

Not applicable.
